# Applying a framework to assess the impact of cardiovascular outcomes improvement research

**DOI:** 10.1186/s12961-021-00710-4

**Published:** 2021-04-21

**Authors:** Mitchell N. Sarkies, Suzanne Robinson, Tom Briffa, Stephen J. Duffy, Mark Nelson, John Beltrame, Louise Cullen, Derek Chew, Julian Smith, David Brieger, Peter Macdonald, Danny Liew, Chris Reid

**Affiliations:** 1 Centre for Healthcare Resilience and Implementation Science, Australian Institute of Health Innovation, Faculty of Medicine, Health and Human Sciences, Macquarie University, 75 Talavera Road, Sydney, NSW 2109 Australia; 2 Health Systems and Health Economics Group, Health Research and Data Analytics Hub, School of Public Health, Faculty of Health Sciences, Curtin University, Perth, Australia; 3 Faculty of Health and Medical Sciences, Population and Public Health, The University of Western Australia, Perth, Australia; 4 Department of General Cardiology, Alfred Health, Melbourne, Australia; 5 Department of Epidemiology and Preventative Medicine, Monash University, Melbourne, Australia; 6 Menzies Institute for Medical Research, University of Tasmania, Hobart, Australia; 7 Discipline of Medicine, University of Adelaide, Adelaide, Australia; 8 Cardiology Department, Central Adelaide Local Health Network, Adelaide, Australia; 9 Cardiology Department, Lyell McEwin Hospital, Adelaide, Australia; 10 Institute of Health and Biomedical Innovation and School of Public Health and Social Work, Faculty of Health, Queensland University of Technology, Brisbane, Australia; 11 Emergency and Trauma Centre, Royal Brisbane and Women’s Hospital, Brisbane, Australia; 12 School of Medicine, Faculty of Health and Behavioural Sciences, The University of Queensland, Brisbane, Australia; 13 Department of Cardiovascular Medicine, Flinders University, Adelaide, Adelaide, Australia; 14 Department of Surgery (School of Clinical Sciences At Monash Health), Monash University, Melbourne, Australia; 15 Department of Cardiothoracic Surgery, Monash Health, Melbourne, Australia; 16 Division of Cardiology, Concord Hospital and University of Sydney, Sydney, Australia; 17 St Vincent’s Hospital, Victor Chang Cardiac Research Institute, University of New South Wales, Sydney, Australia; 18 NHMRC Centre for Research Excellence in Cardiovascular Outcomes Improvement, Health Research and Data Analytics Hub, School of Public Health, Faculty of Health Sciences, Curtin University, Perth, Australia

**Keywords:** Research impact, Impact matrix, Research output, Implementation science, Cardiovascular outcomes, Evaluation, Health research, Research translation, Knowledge translation, Dissemination

## Abstract

**Background:**

Health and medical research funding agencies are increasingly interested in measuring the impact of funded research. We present a research impact case study for the first four years of an Australian National Health and Medical Research Council funded Centre of Research Excellence in Cardiovascular Outcomes Improvement (2016–2020). The primary aim of this paper was to explore the application of a research impact matrix to assess the impact of cardiovascular outcomes improvement research.

**Methods:**

We applied a research impact matrix developed from a systematic review of existing methodological frameworks used to measure research impact. This impact matrix was used as a bespoke tool to identify and understand various research impacts over different time frames. Data sources included a review of existing internal documentation from the research centre and publicly available information sources, informal iterative discussions with 10 centre investigators, and confirmation of information from centre grant and scholarship recipients.

**Results:**

By July 2019, the impact on the short-term research domain category included over 41 direct publications, which were cited over 87 times (median journal impact factor of 2.84). There were over 61 conference presentations, seven PhD candidacies, five new academic collaborations, and six new database linkages conducted. The impact on the mid-term research domain category involved contributions towards the development of a national cardiac registry, cardiovascular guidelines, application for a Medicare Benefits Schedule reimbursement item number, introduction of patient-reported outcome measures into several databases, and the establishment of nine new industry collaborations. Evidence of long-term impacts were described as the development and use of contemporary management for aortic stenosis, a cardiovascular risk prediction model and prevention targets in several data registries, and the establishment of cost-effectiveness for stenting compared to surgery.

**Conclusions:**

We considered the research impact matrix a feasible tool to identify evidence of academic and policy impact in the short- to midterm; however, we experienced challenges in capturing long-term impacts. Cost containment and broader economic impacts represented another difficult area of impact to measure.

**Supplementary Information:**

The online version contains supplementary material available at 10.1186/s12961-021-00710-4.

## Background

Health and medical research funding agencies are increasingly interested in measuring return on investment through improved health outcomes. This focus reflects the ultimate goal of research to generate new knowledge that can be applied to improve health and reduce the burden of illness. Historical investment in health research has not always translated into healthcare policy and practice [1–3], leading to an equivocal use of scarce resources in both the generation of research and functioning of health systems [4, 5]. Consequently, measuring the impact of research including and beyond academic outputs, such as peer-reviewed journal articles, is crucial [6–9]. However, there is a paucity of evidence suggesting an increase in the number of health research projects that measure impact, particularly beyond academic criteria [10].

**Fig. 1:**
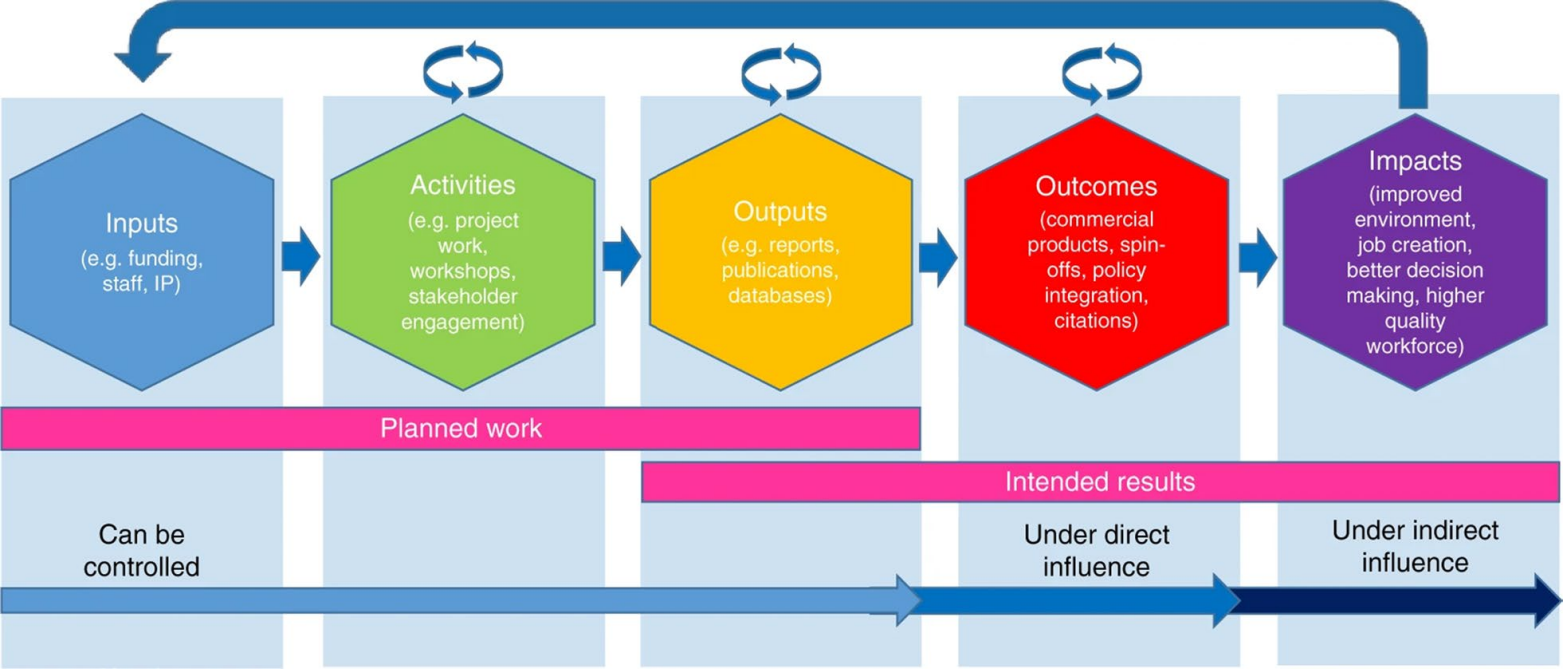
Linear research impact pathways (Source: Fryirs et al. [12], modified from Morgan et al. [15]. Use of this image is supported by the Creative Commons Attribution Non-Commercial (CC BY-NC 4.0) license. See: http://creativecommons.org/licenses/by-nc/4.0/)

Measuring the impact of health research is an evolving and contested activity. The pathway of research impact is often conceptualized and represented as a simple linear process (Fig. 1). However, impacts are rarely manifested through linear pathways in complex systems such as healthcare [11]. This leaves the rigidity of many frameworks for measuring research impact as potentially unfit for this purpose, if they misrepresent the complex research—practice ecosystem. The focus of research policy has also shifted over the years from research utilization to knowledge mobilization [12]. Early tools for the assessment of impact almost solely captured academic productivity, rather than broader impacts to society [13]. In recent years, research impact is increasingly conceptualized in terms of promoting national prosperity, as the focus of research policy has turned toward impacts on the economy, society, environment, and culture; for example, the introduction of a “National Interest Test” in 2018 for Australian research grants allows the Commonwealth Minister for Education veto power to reject applications recommended for funding by the Australian Research Council [14]. Given the conceptual ambiguity of “research impact”, it can be difficult for academics to articulate the patterns and pathways by which transformation of their research into “impact” occurs.

There are numerous ways to define research impact [16]. The International School on Research Impact Assessment, which was set up to facilitate the application of robust and repeatable evaluation methods for research impact, recommends analysis of the research context prior to conducting a research impact assessment. This paper centres on a case study for the evaluation of research impact in cardiovascular outcomes research; therefore, we selected a research impact definition that was considered contextually applicable, defined as “the demonstrable effect from basic, health systems, patient and population-oriented research, and clinical trials that ultimately improve healthcare delivery, human health and quality of life, and generate benefits for the economy, society, culture, public policy or the environment.” [10, 16]. The importance of measuring this impact is clear. Large amounts of public funding are directed towards health research, so it is important that recipients of those funds show accountability and demonstrate relevance to the needs of clinicians, patients, and the health systems within which they operate [17]. Furthermore, the allocation of research funding increasingly relies on information regarding the impact of previous projects.

A range of approaches to measuring research impact have been adopted internationally [18], perhaps most prominently the “Payback” method [19]. Payback measures both academic outputs and wider societal benefits to assess the impact of health research [20]. There are two components to the Payback, one is a model that studies specific components of research and dissemination, and the second is a classification scheme for outputs, outcomes, and impacts. However, the Payback and similar frameworks are generally used to measure research benefit at the macro-scale (e.g. nationally) over long time periods (often decades) and may be cumbersome to apply to smaller, time-constrained programmes of research or individual projects, where specific funding for this additional layer of evaluation may not be available. More recently, Searles et al. developed the Framework to Assess the Impact from Translational (FAIT) health research, which can be feasibly applied to individual research projects. This framework incorporates three approaches: (1) modifications to the Payback Framework to help quantify research impact; (2) an economic measure of social return on investment; and (3) narrative case studies of how the research generated impact, particularly for those that are difficult to quantify [21]. The FAIT relies on two assumptions: (1) that research translation activity has occurred within the life cycle of the project and (2) measurement has been applied prospectively. For projects where these assumptions cannot be met, a pragmatic approach could be considered. Bespoke tools that consider a suite of potentially appropriate metrics drawn from multiple frameworks may allow more consideration of the complex, contextually dependent nature of translating research into impact.

Cardiovascular research provides a good case study for the evaluation of impact, due to the large volume of allocated funding and academic outputs produced. Broadly, the long-term return from cardiovascular research has seen substantial improvements in survival of people affected by cardiovascular disease [22]. Whether new research findings can continue the success in translating findings into improved health outcomes remains an open question. To date, there is a paucity of information documenting the wider impact of specific cardiovascular research projects. Here, we explore the application of a research impact matrix to assess the impact of cardiovascular outcomes improvement research, including both “hard” impacts that can be directly attributed and “soft” impacts indirectly attributed to an Australian collaborative research centre. We present a programme impact evaluation case study for the first four years of a Centre of Research Excellence in Cardiovascular Outcomes Improvement (2016–2020) initiative, funded for AUD$ 2,583,231 by the Australian National Health and Medical Research Council. The Centre of Research Excellence funding scheme provides support for teams of researchers to pursue collaborative research and develop capacity in clinical, health services, public health, and dementia research. The objective of the scheme is to improve health outcomes and promote or improve translation of research outcomes into policy and practice, in addition to supporting researchers in capacity-building activities [23].

We document the feasibility, lessons learned, and challenges encountered when applying a research impact matrix to explore how research output from this centre has influenced healthcare policy and practice. The primary aim of this paper was to explore the application of a research impact matrix to assess the impact of cardiovascular outcomes improvement research.

## Methods

### Case study context

The process of applying a framework to assess the impact of cardiovascular outcomes improvement research is explored through a nationally funded research centre. This centre involved a collaborative team of 10 investigators from multiple academic institutions and health service organizations across Australia. The consortium of research-focused clinicians, academics, consumers, and other stakeholders fostered development of national capability in cardiovascular comparative effectiveness and quality of outcomes research, by utilizing established and developing the footprint of cardiovascular registries. A key aim of the centre was to develop a national platform for encouraging and facilitating excellence in cardiovascular trials, achieved through the establishment of the Australian and New Zealand Alliance for Cardiovascular Trials network. Themes of research for the centre were risk factors, acute cardiovascular disease, coronary artery revascularization procedures (cardiac percutaneous coronary intervention and cardiac surgery), and the management of heart failure. Details of the seed projects and scholarships directly funded by the centre are provided in Additional file 1.

### Selecting a research impact framework

There is currently little consensus on the most appropriate tool to measure research impact. We conducted a literature review to identify a health-focused research impact framework that would meet the specific needs of the key stakeholders, primarily (1) without a requirement for prospectively applied measures and (2) a simple and feasible approach to include different health impact categories and metrics within a single tool. However, we were unable to identify a unique research impact assessment framework meeting the full set of requirements.

Instead, we took a pragmatic approach and considered the application of a research impact matrix developed by Rivera et al. [24]. This research impact matrix was developed from a systematic review of existing methodological frameworks used to measure research impact, summarizing common themes and metrics. It allows the selection of components from existing methodological frameworks, generating a bespoke tool to understand and maximize various impact pathways over different time frames. Rivera et al. identified five major impact categories, 16 impact subgroups, and 80 different metrics across 24 existing methodological frameworks [24]. The five impact categories are (1) primary research-related impact, (2) influence on policy-making, (3) health and health systems impact, (4) health-related and societal impact, and (5) broader economic impact. Researchers do not necessarily need to address every aspect of the methodological framework, as every project is expected to impact the categories and subgroups differently. The original authors suggest a multidimensional approach that adopts narratives, quantitative metrics, and elements from other frameworks, arguably representing a novel and more comprehensive method of impact assessment than reliance on a single tool.

### Choosing the measurement approach and metrics

Impact does not occur at single time points. It can be sporadic or sustained. Rarely will linear pathways adequately capture the complex and contested nature of this process. Therefore, we must acknowledge both the limitation of categorizing certain types of impact based on time frames as well as the pragmatic necessity of doing so. How we represent and measure the impact of cardiovascular outcomes research according to short-, mid- and long-term timelines poses a particular challenge, as in some instances research has taken 25–50 years to deliver impact [25–27]. Impactful research can lead to paradigm shifts that reshape the field and context of application, framing further phases of research, policy and practice. Capturing the entirety of these processes can be challenging, if not impossible.

In light of these challenges, we have outlined how we allocated each measurement and metric to the research impact matrix categories. We did not take a deductive approach of pre-specifying metrics; rather an iterative stance was taken to ensure metrics were contextually relevant to the specific cardiovascular health research undertaken. In the early stages of identifying potentially relevant metrics and measurements of impact, it became clear that mapping and attributing impacts across various programmes of research within an umbrella consortium would be particularly difficult. There were a wide range of both direct and indirect impacts, and uncoupling the centre’s activities from those of its component members or determining the counterfactual of impact that would have occurred without the existence of the centre presented unique challenges. To address these challenges, we attempted to articulate what we considered both “direct” and “indirect” impacts, which were aligned to the notion of “soft” and “hard” impacts used in the River Styles Framework, which is another research impact framework used in the environment sciences [12].

#### Research-related impact

Academic measurements capturing metrics such as knowledge generation, dissemination, and the development of capacity and networks were considered short-term impacts, as the first evidence of return on research investment. Direct research-related impacts were those metrics (e.g. publications) arising from projects funded by the centre’s grants and scholarships. Indirect impacts were metrics associated with the centre, for example, supporting the establishment of a clinical trial network, which was a joint effort of cardiovascular researchers both internal and external to the centre.

#### Influence on policy-making

Metrics that captured the interaction between academics and policy-makers to develop and implement policy were considered mid-term impacts (approximately 1–3 years). These outcomes represented interim impacts that are reliant upon building an evidence base and provide a pathway to translating the benefits of this evidence into practice. Direct influence on policy-making impacts was articulated as metrics with clear attributable links to centre-funded activity, such as publications from centre-funded projects being cited in clinical practice guidelines or centre-funded PhD candidates transitioning to industry, clinical, or academic roles. Indirect impacts were framed as the involvement of centre investigators in policy-making (e.g. contributing to the development of a national cardiovascular registry).

#### Health and health systems impact, health-related and societal impact, and broader economic impact

These impacts represented a time frame beyond five years according to the research impact matrix, requiring a mix of ex-post and ex-ante reporting, as the centre had only been running for four years at the time of research impact evaluation. However, some of the impact subgroups had been captured within the four-year time frame.

### Process

The research impact assessment was conducted in two stages, as described below.

Stage 1: Involved preliminary mapping of the centre impacts according to the matrix of impact categories and subgroups. Review of existing internal centre documentation and publicly available information sources (e.g. Google Scholar) was used to allocate known impacts according to short-, mid- and long-term timelines. Stage 1 was conducted by an independent researcher (MS) who was not involved in any of the projects or running of the centre. The initial research impact matrix was then sent to the centre investigators for their review and amendments, completing the first draft.

Stage 2: Informal semi-structured telephone discussions were then conducted between April and June 2019 by the independent researcher (MS) with each of the centre investigators for every project, grant, and scholarship. These telephone discussions were used to identify the relevant impacts achieved and which impact category and subgroup they best aligned with (approximately 20–60 min). The conversation was structured using the research impact matrix developed by Rivera et al. [24], and questions were asked about each of the impact categories and subgroups. Further prompts were provided for specific example metrics when the centre investigators were uncertain of how to interpret the impact categories and subgroups. Notes were taken by the independent researcher throughout the discussion, which were used to summarize the research impact matrix results for the centre’s projects. Iterative discussions were decided as the best way to elicit different forms of impact, as it allowed the investigators to explore their projects and clarify whether certain measures and metrics could be considered “impact”. This predominantly synchronous exchange was considered a more appropriate approach, as asynchronous communication over email or via a survey was thought to potentially risk constraining the process and lead to missing key impacts. Most of the investigators were located in different states or internationally at the time of the evaluation. One of the investigators could not be interviewed, as they passed away shortly after the centre’s commencement. Upon completion of the telephone discussions with each investigator, additional impacts were sought by emailing the completed impact matrix to each of the centre’s grant and scholarship recipients to confirm the information obtained and add anything missed during the discussions with centre investigators. These grant and scholarship recipients were mostly early career researchers who were able to provide additional contextual information regarding the projects and research impact. A manual search of academic outputs was then performed to obtain citations and impact factors for the identified publications.

## Results

### Case study: Centre for Research Excellence in Cardiovascular Outcomes Improvement

Over the first four years of the research centre, there were 13 seed grant projects, seven scholarships, and three investigator-led projects. Seed grant projects typically funded early career researcher-led investigations under the supervision of one or more of the centre’s investigators. The scholarships were directed towards PhD candidates who were considered to have high potential to attract additional sources of funding for their work. Three investigator-led projects encompassed larger initiatives, which were part of broader efforts outside the centre. Scholarship recipients were expected to obtain further funding from national and other sources to free up scholarship funding for more researchers, which was achieved throughout the life of the centre. The research impact matrix results for the centre’s projects are presented in Table 1.

**Table 1:** The research impact matrix for the Centre for Research Excellence in Cardiovascular Outcomes Improvement projects

Time frame	Impact categories	Impact subgroups	Outputs/outcomes
Short-term	1. Research-related impact	Research and innovation outcomes	Direct Peer-reviewed publications (*n* = 41) Number of citations (*n* = 87) Journal impact factor (median 2.843; range 1.248 to 23.239) Manuscripts under review or development (*n* = 26)
			Indirect Associated publications (*n* = 156)
		Dissemination and knowledge transfer	Direct Conference presentations (*n* = 61) Total PhD candidacies commenced (*n* = 7): PhD candidacies with cross-institutional collaboration (*n* = 1)
		Academic collaborations, research networks and data sharing	Direct Total new academic collaborations (*n* = 5): International collaborations (*n* = 3) New database linkages (*n* = 6): Admitted episodes & emergency episode National Death Index Ambulance Victoria Australian and New Zealand Society of Cardiac and Thoracic Surgeons National database Combined Australian acute coronary syndrome registries (*n* = 16,500) Development of a multistate linked data platform for analysis of coronary heart disease in younger adults
			Indirect Utilization and reinforcement of a national general practice network to conduct research (> 2000 members) Establishment of the Australian and New Zealand Alliance for Cardiovascular Trials network (> 200 members)
Mid-term	2. Influencing and involvement in policy-making	Level of policy-making	Indirect Centre contribution to the development of a national cardiac data registry Centre contribution to the development of the National Heart Foundation guidelines [35-37]
		Type and nature of policy impact	Direct Contribution to Medicare Benefits Schedule reimbursement item number application: Ambulatory blood pressure monitoring Implementation of patient reported outcome measures in several data registries: Coronary Angiogram Database of South Australia Victorian Cardiac Outcomes Registry
		Policy networks	Direct Establishment of new industry collaborations (*n* = 9) Embedded academic and statistician roles in industry Western Australian Department of Health PhD candidate transition to industry role (*n* = 1)
Long-term	3. Health and health systems impact	Evidence-based practice	Direct Establishment of longitudinal data capture regarding change in practice: Coronary Angiogram Database of South Australia [38] Percutaneous coronary intervention [39] Door-to-balloon time [40] Coronary artery bypass graft surgery [41] Development of contemporary management for aortic stenosis [42, 43] Development of a risk prediction model linked with cardiovascular data registries, which is reportedly used ongoing and has directly impacted hospital care [44, 45]
		Quality of care and service delivery	Direct Validation and auditing of registry data so it can be used to improve quality of care and service delivery: Coronary Angiogram Database of South Australia Melbourne Interventional Group database [46] Australasian Society of Cardiac and Thoracic Surgeons database [46] Prevention targets developed from the identification of risk factor burden, clinical profile, and morbidity patterns of adults < 55 years with acute coronary syndrome
		Cost containment and effectiveness	Direct Cost-effectiveness of surgery vs stenting established: Percutaneous coronary intervention vs surgery for the treatment of multivessel coronary artery disease in the drug-eluting stent era [47] Coronary artery bypass surgery vs stenting in high-risk patients [48] Guideline-driven use of drug-eluting stents [49]
		Resource allocation	No information identified
		Health workforce	No information identified
	4. Health-related and societal impacts	Health literacy	No information identified
		Health knowledge, attitudes and behaviours	No information identified
		Improved social equity, inclusion or cohesion	No information identified
	5. Broader economic impacts	Economic impacts	Direct PhD candidates transitioned from centre-funded to externally funded scholarship (*n* = 6; AUD$300,586) Centre grant recipients awarded additional external research funding: National Health and Medical Research Council (total AUD$2,481,816) Medical Research Future Fund (total AUD$2,062,697) Leveraged industry grants (*n* = 2): Emergency Medicine Research Foundation (AUD$150,000) National Heart Foundation Vanguard Grant (AUD$73,000)

The term industry refers to all non-academic organizations, including governmental, non-governmental, and private

The centre covered short-term impacts across research and innovation outcomes, dissemination and knowledge transfer, academic collaborations, research networks, and data sharing. The use of prompts by the independent researcher elicited previously unreported impact metrics, allowing quantitative measurement. Mid-term impacts were captured under one category of influencing and involvement in policy-making, with three subgroups: level of policy-making, type and nature of policy impact, and policy networks. While these impacts were known to the investigators, they had not been explicitly measured as research impacts elsewhere. Descriptions of these impacts were difficult to quantify, such as contributions to establishment of registries, guideline development, and Medicare Benefits Scheme reimbursement item number applications. Long-term impacts were reported under three categories, across nine subgroups. Impact for some subgroups were unable to be identified due to the formative nature and preliminary timing of this impact evaluation. A mix of descriptive and quantitative measurements were reported for evidence of change in clinical practice, patient outcomes, consumer involvement, and further research funding.

### Feasibility of the research impact matrix

We considered the research impact matrix a feasible tool to identify evidence of academic and policy impact across short- and mid-term timeframes, otherwise not documented through traditional research criterion and project evaluations. We did not identify any types of impact that could not be categorized into the research impact matrix domains. For the centre’s projects, the matrix assisted the investigators in contemplating outputs across the range of categories and subgroups that otherwise would have gone undocumented. Support and prompting from the independent researcher were crucial to the identification of these outputs and impact, as well as describing and quantifying metrics for each domain. Given the importance of this synchronous exchange, we remain convinced that relying on the centre documentation and publicly available information alone would not have been sufficient to capture the full range of impacts. Further, an asynchronous approach using survey or email exchange would likely not have resulted in the same depth of exploration with the centre’s investigators. Most of the reported research impact was captured under the short-term research domain category, such as scientific journal publications, citations of published work, dissemination of information and knowledge transfer (e.g. conference presentations), and academic collaboration activity. The most challenging areas of impact to measure were related to long-term health and health systems, health-related and societal, and broader economic outcomes.

### Challenges encountered when applying the research impact matrix

The impact categories considered within the long-term time frame were the most challenging aspects to measure and report. Measuring health and health system impact through changes in evidence-based practice would require fundamentally different study designs to those typically employed in clinical research projects. Most of the funded work within the centre was engaged in effectiveness research, focused on translating the benefits of therapies from efficacy trials to real-world settings. However, an entirely different set of study designs is required to enable changes in evidence-based practice, namely encompassed within the field of implementation science. Implementation studies are positioned at the end of the research translation pipeline, and typically require the evidence base for an intervention, programme, or policy to be established prior to exploring how it may be implemented into practice [28, 29]. The time frames for this process are typically beyond what would be considered feasible for an ex post research impact evaluation of this nature, given it reportedly takes, on average, approximately 17 years for only 14% of research into practice [30].

Changes in health-related societal impacts were also difficult to measure given the first four-year time frame of the centre’s work. Capturing broader societal changes in health literacy, knowledge, attitudes, and behaviours proved beyond much of the data captured by clinical research translation activities. The paucity of available data in these areas of research impact indicates that prospective planning and data collection may be warranted for other projects wishing to capture these categories of research impact.

Cost containment and broader economic impacts represented another difficult area of impact to measure. The data needed to model economic returns was not always adequately captured. Further, input from a health economist, while available for our work, is not always resourced in project teams.

## Discussion

### Lessons learned from the application of a research impact matrix

In applying a research impact matrix for a case study in cardiovascular outcomes improvement it became clear that there is no one tool (or set of tools) that can cover the diversity of circumstances in health research. The complexity of capturing all relevant measures of impact not only presented itself when trying to choose a framework, but became more apparent when identifying and capturing potential metrics as well as categorizing these according to timeframes and domains of impact. On reflection, it was important to tailor the choice of framework and measures of impact iteratively throughout the process, particularly given the cyclical nature of inputs, activities, outputs, and outcomes in research which is striving to generate new knowledge.

An important lesson to arise from our application of a research impact framework is the predominantly context-dependent nature of research impact [16, 31]. The presented case study is situated in a mixed public and private funded health system from a high-income country, where impact is typically conceptualized as a direct and linear process from quantitative findings to relatively short-term changes one step removed from patient outcomes [18]. Our case study unsurprisingly favoured a similar narrative, focusing on potentially high economic returns without in-depth exploration of the interactions by which indirect impacts might occur. Methods that incorporate co-design principles, participatory designs, and rigorous qualitative approaches might have allowed us to capture the nature and mechanisms of research impact, potentially creating a more complete understanding of this multidimensional process within complex health systems.

Another important learning from this work pertains to the respective value and benefit of a formative or summative approach to research impact. We took a formative approach exploring the research impact on cardiovascular outcomes improvement ex-post from the first four years of the collaborative centre. This was done to ensure that findings could be used by the investigators to inform ongoing decisions regarding the investment of research funding towards implementation and translation activities, before the end of the funding period. Four years is a relatively short time period in cardiovascular outcomes research [16]. However, anecdotal feedback from the centre’s investigators indicated that exploring their research impact formatively encouraged prospective investment of resources in research translation projects and activities. The centre investigators often articulated how their research might achieve impact ex-ante, through the discussions when applying the research impact matrix to their projects. The formative application of impact frameworks to encourage research translation has been proposed by Ramanathan et al., which provides the additional benefit of facilitating collection of process, output, and interim outcomes related to research impact [10].

### Suggestions for future research impact assessment in cardiovascular outcomes research

In the presented case study, we applied the research impact matrix retrospectively to the first four years of the collaborative centre, identifying mostly the ex-post impacts of the centre. We propose that a prospective approach from the beginning of the funding period (including prospective data collection) may have yielded more insightful findings, as relevant measurements could have been preplanned to capture the expected research impacts over the lifetime of the funded centre. We were unable to capture many important metrics or the requisite data to conduct an economic impact evaluation. Further, articulating ex-ante pathways for research impact at the beginning of the case study projects may have promoted additional translation- and implementation-focused designs to be incorporated with the studies on the effectiveness of different treatment modalities for cardiovascular outcomes improvement. Future evaluations of the impact of research may provide a more accurate, nuanced, and useful evaluation if prospective planning can take place prior to awarding of research funding. If appropriate measures can be identified and collected for both formative and summative evaluation, research activity may be able to be more targeted towards translation and impact, particularly at the later periods of funded projects. Research funding agencies may be well served by requiring reporting of the impact and translation of funded research projects to ensure future awarding decisions are made to groups with a track record of translating and implementing research into practice [32].

### Strengths and limitations

One of the main strengths of this study was the pragmatic approach to apply a research impact matrix that summarized common themes and metrics used across several existing methodological frameworks. This approach had two main benefits; firstly, it allowed the sufficient tailoring to the context of application where specific measures of impact were not collected prospectively, and secondly, provided a simple, contemporaneous, and resource-efficient process to identify different impact categories across the programme of research. The time and cost involved in applying many research impact assessment frameworks is an important consideration for research teams and funding agencies, given the potential opportunity costs associated with their implementation. Opportunity costs occur because resources spent on conducting a research impact assessment are the same resources that can no longer be used to conduct otherwise valuable research [33]. Therefore, it is important to ensure the benefits of research impact assessment outweigh the costs of engaging in this activity [34]. Given the pragmatic nature of our approach, it was important to ensure robustness and trustworthiness. Multiple data sources, including documentation review, bibliometric searches, and discussions with the centre investigators, were used to triangulate information ideally ensuring multiple accounts pointed to the same result. An independent researcher collated the information and engaged in the discussions with centre investigators, providing a validating check to ensure suggested outputs and impact were objective, administratively efficient to apply metrics, transparent, and comparable. Further, claims made by the centre investigators were confirmed with scholarship and grant recipients as an additional source of validation.

There were limitations to conducting the research impact evaluation before the completion of the centre’s funding term. The impact of research usually takes a long time to occur, often well beyond the completion and dissemination of the findings. The relatively short time frame of four years since research programme commencement meant that it was difficult to capture measures of impact beyond short-term academic outputs and mid-term influence on health policy. Reliance on retrospective, self-reported recall from investigators also limited the ability to apply quantitative measures and metrics for several of the reported impacts. This limitation resulted in the reliance on descriptions, which are difficult to verify. Further, a more robust qualitative approach could have been used to obtain more trustworthy responses during the semi-structured discussions with centre investigators. Some research impact evaluations include an exploration of the economic benefits of the research conducted, but this was not completed as part of our evaluation due to its formative nature and limited prospectively collected data regarding cost-effectiveness. Our limited exploration of the activities and pathways to research impact constrained the ability to trace a linear logic from “the bench to bedside”. This was perhaps a result of the research impact matrix selected and focus of the relevant stakeholders’ needs. Arguably, a more in-depth exploration of the pathways to impact may have elucidated where to best direct research translation investment, but this was beyond the scope of our project. While some efforts were taken to verify the claimed impacts from the centre’s research, much of the data collection was reliant on self-reported measures. This reliance brought about a tension between attribution and contribution, making it difficult to attribute certain impacts to particular studies, especially when much of the centre’s work was incremental and collaborative. However, unlike the scientific approach which aims to produce valid, reliable, and generalizable findings, the exploration of research impact seeks to understand how research has led to impact from the perspectives of specific stakeholders [34].

## Conclusion

The application of a bespoke research impact matrix uncovered the impacts of cardiovascular outcomes improvement research across and range of domains and timeframes. We did not identify any types of impact that could not be categorized into the research impact matrix domains. Using both synchronous and asynchronous means of capturing data from multiple sources required support and prompting from the independent researcher but led to identification of impact that had not otherwise been captured via traditional criterion and evaluations. We considered the research impact matrix a feasible tool to identify evidence of academic and policy impact in the short- to mid-term; however, we experienced challenges in capturing long-term impacts of cardiovascular outcomes improvement research. Cost containment and broader economic impacts represented another difficult area of impact to measure.

## Supplementary Information

**Additional file 1: Appendix S1.** Seed grants and scholarships directly funded by the CRE in cardiovascular outcomes improvement.

## Data Availability

Data are available from the corresponding author on reasonable request.

## Abbreviations

FAIT: Framework to Assess the Impact from Translational health research
