# Supplementary material for: Applying a framework to assess the impact of cardiovascular outcomes improvement research
Source: Health Res Policy Syst. 2021 Apr 21;19:67. doi: 10.1186/s12961-021-00710-4 (PMC8059028; doi:10.1186/s12961-021-00710-4)
Supplement: Supplementary file 1 — Additional file 1: Appendix S1. Seed grants and scholarships directly funded by the CRE in cardiovascular outcomes improvement. [file 12961_2021_710_MOESM1_ESM.docx]

Appendix 1. Seed grants and scholarships directly funded by the CRE in cardiovascular outcomes improvement

| **Title** | **Aim** | **Synopsis** | **Funding** | **Year** |
| --- | --- | --- | --- | --- |
| **Seed funding** | | | | |
| ANBP2 cohort study analysis: long-term outcomes for treated elderly hypertension patients | Determine clinical factors associated with maintaining health active life in an older treated hypertensive population | Analysis of long-term outcomes (>10 years) from patients >65 years in the 2nd Australian National Blood Pressure Study demonstrating the effects of ACE inhibitors versus diuretics in the treatment of high blood pressure | AUD 25,000 | 2017 |
| SNAPSHOT-AF: who gets stroke prevention for atrial fibrillation in the emergency department? Use of oral anticoagulation in the emergency department setting in Australia and New Zealand | Determine whether patients presenting to emergency with an irregular heart rhythm (AF) receive appropriate blood-thinning treatment to prevent stroke when they are discharged | Identification of current practice in terms of oral anticoagulant use for stroke prevention in patients seen in the emergency department, with reference to use of novel oral anticoagulants compared to warfarin and potential gaps between contemporary guidelines and current practice in Australia and New Zealand | AUD 25,000 | 2017 |
| Automated data extraction of electronic health records from emergency department presentations using natural language processing for a state-wide chest pain clinical data registry: The HER DECoDeR Study | Develop a computer application to automatically retrieve relevant information from electronic health records of emergency department patients who present with chest pain | Utilising natural language processing technology to enhance collection of clinical data and lower overhead costs. This is the first instance to use this technology to enhance data collection for an emergency medicine clinical database in Australia | AUD 25,000 | 2017 |
| Data linkage with the Victorian Emergency Minimum Dataset, Victorian Cardiac Outcomes Registry and the ANZSCTS databases | Overall, the aim of the data linkage with the Victorian Admitted Episodes Dataset is to enable examining of the outcomes of patients in the Victorian Cardiac Outcomes Registry and ANZSCTS database | Further enhance the utility of the cardiac registry data. This allows for assessment of readmissions and health service encounters and provide valuable information as to the outcomes of patients entered into the registries beyond the current 30 day follow-up currently undertaken | AUD 25,000 | 2017 |
| Statistical services support to enable advanced analysis of the Melbourne Interventional Group and Victorian Cardiac Outcomes Registry percutaneous coronary intervention data | Provide statistical services to Melbourne Interventional Group and Victorian Cardiac Outcomes Registry participating hospitals | All Melbourne Intervention Group participating hospitals receive statistical support to support and mentor junior doctors engaged in research | AUD 25,000 | 2017 |
| Collection of formative data for an Australian-led international trial randomising patients with severe but asymptomatic aortic stenosis to a conventional strategy of ‘watchful waiting’ or to aortic valve replacement | Derive pilot data to inform the design of a RCT assessing the relative risks and benefits of early aortic valve replacement versus watchful waiting in patients with severe asymptomatic aortic stenosis | Formative project to provide crucial pilot data, together with gaining ethics approvals, and establishing a research network for external research funding grant applications | AUD 25,000 | 2017 |
| Detailed analyses of data from the Victorian Cardiac Outcomes Registry linked to the Victorian Admitted Episodes Dataset, the Victorian Emergency Minimum Dataset and the National Death Index | Capture long-term hospital and mortality outcomes among Victorian Cardiac Outcomes Registry subjects and determine predictors of these outcomes | Assessment of long-term outcomes for Victorian Cardiac Outcomes Registry patients, including factors associated with long term mortality and morbidity | AUD 25,000 | 2018 |
| Advanced analyses of the Melbourne Interventional Group percutaneous coronary intervention registry data | Provide statistical services to Melbourne Intervention Group participating clinicians and hospitals for research purposes | All Melbourne Interventional Group participating hospitals receive statistical support to support and mentor junior doctors engaged in research and quality assurance activities at participating percutaneous coronary intervention hospitals | AUD 25,000 | 2018 |
| CAPLA - An international multi centre randomised controlled trial to evaluate the long-term outcomes of two atrial fibrillation ablation techniques | Evaluate outcomes of atrial fibrillation ablation in patients using two strategies (pulmonary vein isolation or posterior wall isolation) in 334 patients at 12 months | International multi centre randomised controlled trial to evaluate the long term outcomes of two atrial fibrillation ablation techniques (pulmonary vein isolation vs posterior wall isolation in addition) in patients with persistent atrial fibrillation undergoing atrial fibrillation ablation | AUD 25,000 | 2018 |
| Coronary heart disease in men and women aged under 55 years: developing an evidence base for improving prevention and management | Develop a big-data platform of multi-state linked coronary heart disease and acute coronary syndrome registry data, for investigation of the coronary heart disease burden in people aged <55 years. | Develop a large linked data platform to provide data on whether incidence rates of heart attacks are worsening, and determine the rate of recurrence and long-term survival in people <55 years in Australia. The data platform incorporates datasets from other jurisdictions and sources as an ongoing monitoring and research tool | AUD 25,000 | 2018 |
| Evaluating the cost-effectiveness of revascularisation strategies in patients with multi-vessel coronary artery disease in Australia | Compare the cost-effectiveness of different intervention strategies in patients with multi-vessel coronary artery disease in Western Australia | In patients with heart attack, the treatment of more than one blocked artery on the heart was previously treatment by open heart surgery. The introduction and widespread uptake of stents to treat blocked heart arteries has provided a new option that does not involve surgery | AUD 25,000 | 2018 |
| A comparison of percutaneous coronary intervention service provision between single-payer and multi-payer health care system; data from the ANZAC-QI and the Victorian Cardiac Outcomes Registry | Describe differences in key aspects of percutaneous coronary intervention service provision between NZ and Victoria, Australia by analysing the nationwide (ANZACS-QI) and state-wide registries | It was hypothesised that Australia and New Zealand differ in percutaneous coronary intervention service provision due to different funding systems. This hypothesis was explored by merging and comparing data registries | AUD 25,000 | 2018 |
| Aspirin as an effect modifier for the effects of exposure to particulate matter and the oxides of nitrogen | Investigate whether particulate matter and nitrogen oxides are associated with cardiovascular disease, diabetes and mental health/ cognitive decline, and whether low-dose aspirin modifies these effects. | First study to investigate whether low dose enteric non-steroidal anti-inflammatory modifies the harmful effects of particulate matter and oxides of nitrogen exposure in Australia and the United States | AUD 25,000 | 2018 |
| **Scholarships** | | | | |
| Towards a multi-marker model of cardiovascular risk – what can pooled analyses of clinical and governmental databases and registries provide in an era of big data? | Explore clinical, metabolic and blood-based biomarkers as determinants of cardiovascular outcomes and the potential utility of these in multimarker models of cardiovascular risk | Strategies for predicting cardiovascular risk are variably effective for predicting response to and guiding intensity of medical and interventional therapies. Pooled large clinical datasets, governmental data sets and registries can better refine these risk models using "big data" approaches | AUD 20,000 | 2017 |
| Optimizing post cardiac arrest myocardial dysfunction with early coronary intervention and assessment by magnetic resonance imaging, invasive coronary hemodynamic measurements and markers of inflammation | Combination of clinical and epidemiological studies to assess the role of cardiac intervention in patients with cardiac emergencies of heart attack and cardiac arrest | Optimizing post- resuscitation care has been shown to be associated with improved patient outcomes. This research assesses the safety and effectiveness of emergent coronary angiography in a randomized population of post- out-of-hospital cardiac arrest patients without ECG changes | AUD 20,000 | 2017 |
| A retrospective comparison of 30-day major adverse cardiac events of patients presenting with chest pain one year before and after implementation of accelerated chest pain risk evaluation project across Queensland Health Emergency Departments: Results from a state-wide chest pain clinical data registry using automated data extraction from electronic health records | This study evaluates clinically important outcomes of emergency patients with chest pain before and after implementation of new guidelines for chest pain assessment | A two hour accelerated diagnostic pathway has since been implemented across 19 Queensland Health emergency departments since 2012. This study reviewed the incidence of 30-day Major Adverse Cardiac Events of emergency patients presenting with chest pain one year before and after implementation of the two hour pathway | AUD 60,000 | 2017 |
| The drivers of readmissions for major cardiovascular conditions and the associated economic impact | Investigate the reasons behind readmissions to hospital across major cardiovascular conditions, and to describe the associated economic impact | Linked administrative records used to determine total hospitalisation and readmission trends for heart failure, atrial fibrillation, acute coronary syndrome and stroke, and model their associated cost | AUD 30,000 | 2018 |
| RCT - STALL-HFpEF: Studies of Atrial Fibrillation and Left Ventricular Remodelling in Heart Failure with Preserved Ejection Fraction | Evaluate peak exercise pulmonary capillary wedge pressure, VO2 max, cardiac MRI (T1 time), echo parameters in patients with AF- heart failure with preserved ejection fraction and the effect of atrial fibrillation ablation at six months | Multi centre randomised trial evaluating medical management vs atrial fibrillation ablation in patients with heart failure with preserved ejection fraction and persistent atrial fibrillation. These are two major epidemics in cardiovascular disease which often coexist with increased associated mortality and healthcare burden | AUD 10,000 | 2018 |
| Microcirculation in ischaemic and non-ischaemic cardiac disease | Evaluate the protective effects of doxycycline on heart muscle in patients with heart attacks when given intravenously prior to opening the infarction-related artery | This study investigates the effect of intravenous doxycycline, a commonly used antibiotic, on reducing heart muscle injury when given before opening an occluded coronary artery that is causing a major heart attack Patients will be assessed with the gold-standard cardiac magnetic resonance imaging to determine the amount of heart muscle damage and heart function. Doxycycline is an inexpensive antibiotic that has numerous other beneficial effects, which might make it useful as additional therapy for the emergent treatment of patients presenting with heart attacks | AUD 30,000 | 2018 |
| Associations between domestic indoor air quality and cardiometabolic risk factors | Investigate potential associations between measured domestic indoor air quality and selected cardiometabolic risk factors in middle-aged adults | Investigation of putative associations between selected cardiometabolic risk factors and measured indoor air pollution in domestic homes. The primary outcome examines the relationship between IAQ and nocturnal blood pressure. Based on the results of this research, a more thorough understanding of the relationship between domestic indoor air quality, and selected functional intermediate outcomes related to cardiometabolic risk will be established | AUD 10,000 | 2018 |

* ANBP2 (Second Australian National Blood Pressure Study); ACE (Angiotensin-converting-enzyme); ANZSCTS (Australian and New Zealand Society of Cardiac and Thoracic Surgeons); RCT (randomised controlled trial); CAPLA (Catheter Ablation for persistent atrial fibrillation); ANZAC-QI (New Zealand Acute Coronary Syndrome Quality Improvement).
